# The Potential Use of Plant Natural Products and Plant Extracts with Antioxidant Properties for the Prevention/Treatment of Neurodegenerative Diseases: In Vitro, In Vivo and Clinical Trials

**DOI:** 10.3390/molecules23123283

**Published:** 2018-12-11

**Authors:** Franziska Pohl, Paul Kong Thoo Lin

**Affiliations:** School of Pharmacy and Life Sciences, Robert Gordon University, Aberdeen AB10 7GJ, UK; f.pohl@rgu.ac.uk

**Keywords:** antioxidants, natural products, in vitro, in vivo, clinical trials, plant extracts, phytochemicals, phenolics, Ginkgo biloba, secondary metabolites

## Abstract

Neurodegenerative disorders, including Alzheimer’s disease, Parkinson’s disease and Huntington’s disease, present a major health issue and financial burden for health care systems around the world. The impact of these diseases will further increase over the next decades due to increasing life expectancies. No cure is currently available for the treatment of these conditions; only drugs, which merely alleviate the symptoms. Oxidative stress has long been associated with neurodegeneration, whether as a cause or as part of the downstream results caused by other factors. Thus, the use of antioxidants to counter cellular oxidative stress within the nervous system has been suggested as a potential treatment option for neurological disorders. Over the last decade, significant research has focused on the potential use of natural antioxidants to target oxidative stress. However, clinical trial results have lacked success for the treatment of patients with neurological disorders. The knowledge that natural extracts show other positive molecular activities in addition to antioxidant activity, however, has led to further research of natural extracts for their potential use as prevention or treatment/management of neurodegenerative diseases. This review will cover several in vitro and in vivo research studies, as well as clinical trials, and highlight the potential of natural antioxidants.

## 1. Introduction

This review aims to give an overview on the importance of oxidative stress and its relevance in neurodegenerative disease. One option for counteracting oxidative stress is the application of natural products obtained from plant extracts. These have been thoroughly tested in vitro (chemical antioxidant activity and cell systems) and in vivo (animal disease models) and have shown promising results. However, results from clinical trial studies have been less successful. Here, recent research on natural extracts, and their potential pitfalls in clinical trials, are discussed.

## 2. Neurodegenerative Disease

Neurodegenerative disease is a heterogeneous group of disorders that are caused by the degradation and subsequent loss of neurons. These changes in the human brain can lead to cognitive or functional decline of the patient over time. The question as to why some people develop neurodegenerative disease and some do not has only partially been answered. While some neurodegenerative diseases can be due to genetic mutations, some are also associated with hazardous living environments. However, some of the causes are still unknown. This is why it is important to (i) study the reasons behind these conditions, and (ii) run drug trials of compounds that might have potential to cure, prevent, or at least delay the onset of neurodegenerative diseases [1].

### 2.1. A Burden on Health Organizations without Current Cure

Neurodegenerative disorders, including Alzheimer’s disease (AD), Parkinson’s disease (PD) and Huntington’s disease (HD), present a major health and financial burden to every health service organization in the world. Due to extended health research and better living conditions in general, the average human life span has increased by about 30 years in the 20th century in countries like USA, Canada, Australia, New Zealand and western Europe [2]. Although this is seen as a great accomplishment, it comes as a double-edged sword, since those diseases most common in the older generation (late onset) are also on the rise. Since many neurological disorders are late-onset diseases, their occurrence will rise in very large numbers over the next century. For example, the total number of AD patients globally is estimated to be well over 100 million by 2050 [3]. However, AD is not the only disease for which the numbers will increase. A paper by Dorsey et al., “Projected number of people with Parkinson’s disease in the most populous nations, 2005 through 2030”, shows similar results. The number of PD patients will double or even increase above that for countries such as China, India and Indonesia [4].

Just like for AD, there is no cure or prevention available for Parkinson’s patients; only the symptoms can be alleviated via drugs treatment or surgery. The lack of knowledge and research in this area has resulted in the slow drug development for neurological research. When the list of 1355 drugs newly approved between 1981 and 2010 [5] is considered, it is interesting to note that anticancer compounds, no matter whether the drug is from natural origins or synthetic (128), are the highest in number, followed by antibacterial (118), antiviral (110) and antihypertensive (79) drugs, in that order. For neurological disease, however, the numbers are much lower. There have been 12 anti-Parkinsonian drugs, 6 drugs for multiple sclerosis, and only 4 anti-Alzheimer drugs in the 30 years of research [5]. It is important to note that none of the so-called “anti-Alzheimer drugs” slow down or prevent neuronal death and the malfunction of the human brain [6]. Pharmacological treatments are only available for partial treatment of the symptoms [7]. No new AD drugs have been approved by the Food and Drug Administration (FDA) since 2003 [8]. This shows the severe lack of knowledge in neurodegenerative disease and demonstrates the requirement of further urgent research in this field. Although research has been progressing well in the field of neurosciences, drug development is falling well behind. The only advances since then have been the addition of a combination therapy using the previously approved drugs memantine hydrochloride (extended-release) and donepezil hydrochloride as Namzaric in 2014 [9]. Since many neurodegenerative diseases are multifactorial, disease combination therapy using a mixture of compounds or for example plant extracts, acting on several disease targets, could lead to promising results [10].

The numbers of drugs approved for other neurodegenerative diseases has not seen much of an increase over the last 8 years either. There was only a single new drug approved for amyotrophic lateral sclerosis (ALS) (Radicava, 2017) and HD (Austedo, 2017). A slightly higher number of drugs has been approved for PD (Duopa, 2015; Gocovri, 2017; Nuplazid, 2016; Rytary, 2015 and Xadago, 2017) [11]. For other neurodegenerative diseases, such as Prion diseases, e.g., Creutzfeldt-Jakob disease, or Spinocerebellar ataxia (SCA), e.g., SCA 1, 3 or 6, no drug therapies to retard their progress is currently available [12]. Treatment options for the latter diseases usually include physical or speech therapy or treatments aiming to alleviate the symptoms and most often requires an approach individualized for each patient. To find additional treatment options, current and new drug targets are under constant investigation.

### 2.2. Current and Future Drug Targets

**Figure 1:**
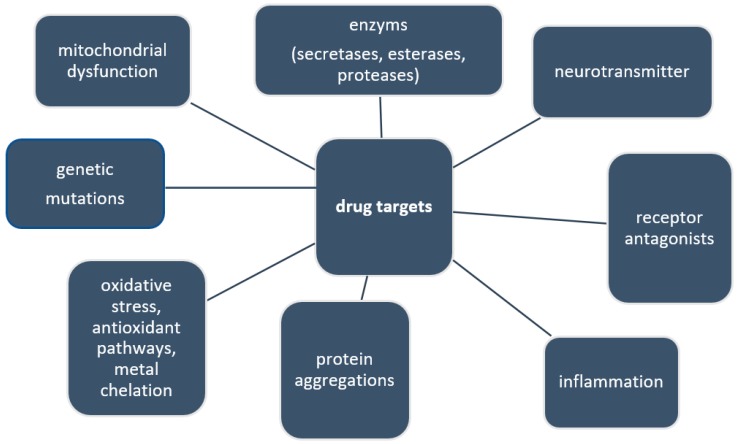
Examples of drug targets for neurodegeneration.

Current research has been focusing on a wide variety of different potential drug targets. In AD they are thought to be, for example, *N*-Methyl-d-aspartate (NMDA) receptor antagonists [13,14], acetylcholinesterase inhibitors [14,15], antioxidants [16], radical scavengers [17], monoamine oxidase inhibitors [15] and Aβ and tau aggregation inhibitors/dissolver [7]. In PD current treatment is mainly based on levodopa, a pre-stage of dopamine production, which can be converted into dopamine by the body to replenish the lost dopamine by the degeneration of dopaminergic neurons, which causes the disease. However, just like for most neurodegenerative diseases, this has helped to alleviate some of the symptoms associated with PD, but neither cures nor halts the disease in any way. Some of the current and future drug targets are summarized in Figure 1.

More recently, the inhibition of asparagine endopeptidase (AEP, a protease responsible for the cleavage of its substrates after asparagine residues) with small molecular inhibitors has been suggested as a potential target for AD, because it is thought to play a role in the pathological processing of the amyloid precursor protein and tau proteins. If the role of AEP in AD brains can be further elucidated, its inhibition could be of importance for other age-related neurological disease such as PD, ALS and frontotemporal lobar degeneration (FTLD) [18].

Also, protein aggregations and their precursor stages have long been the target of several drug trials and significant research, for example AD, PD, and ALS, as well as HD and other polyglutamine disease (e.g., SCA) [19]. New pathways for restoring physiological protein conformations are still commonly sought after. One example being a recent study by Zunke et al. on the “Reversible Conformational Conversion of α-Synuclein Toxic Assemblies by Glucosylceramide”, where the reduction of glycosphingolipid diminished PD pathology in patient-derived neurons [20].

Other new ways of treatment, which have only become a potential option in the last few years, are stem cell gene therapies. Stem cells have the unique property of being self-renewing and can differentiate into specialized cell types. As a result, these have brought a significant amount of attention, especially in the field of neurodegeneration [21]. Studies on these systems have been conducted in vitro and in vivo, as well as in clinical trials in patients. A small Phase I/II and IIa clinical trial on the safety and clinical effects of mesenchymal stem cells secreting neurotrophic factor in patients with ALS found them to be safe and indicated possible clinical effects [22]. In another example, a small phase I study, gene therapy (ex vivo nerve growth factor (NGF) gene delivery) was used on 8 patients with mild AD. Participants had genetically modified autologous fibroblasts, modified to express human NGF, implanted into their forebrain. Follow up checks showed significant improvements in cognitive activities (Mini–Mental State Examination), suggesting the role of genetic modification [23].

Aging, one of the risk factors of many neurodegenerative diseases, was first associated with free radicals by Harman, in “Aging: A theory based on free radical and radiation chemistry” in 1956 [24]. Since then, reactive oxygen species (ROS) have been shown to be the cause of oxidative stress and have been associated with a range of neurological disorders such as AD, PD, HD and Pick’s disease. It is assumed that reducing oxidative stress within the human body could at least be part of future treatment options, targeting multiple drug targets, as most neurodegenerative diseases have been associated with several different pathways involved in the disease development [25,26].

Some of the compounds currently used for the treatment of neurodegenerative disease are of natural origin, such as Levodopa (L-DOPA), currently used for the treatment of PD. Originally, L-DOPA was isolated from seedlings of *Vicia faba* (1910–13) [27]. Similarly, Galantamine hydrobromide, a natural product from plants of the *Galanthus* genus, is used for the treatment of AD [28].

### 2.3. Oxidative Stress

Oxidative stress, a condition where the balance of reactive oxygen (ROS) and reactive nitrogen species (RNS) to antioxidants is in favor of ROS/RNS, is one of the causes associated with many neurodegenerative diseases. ROS, such as superoxide (O_2_^●-^), hydrogen peroxide (H_2_O_2_) and hydroxyl radical (OH^●^), are generated during normal aerobic respiration of the cells. Other sources of oxidants include bacteria- or virus-infected cells that are destroyed by phagocytosis, generation of by-products in peroxisomes (lipid and fatty acid degradation), and by-products generated by cytochrome P450 [16].

**Figure 2:**
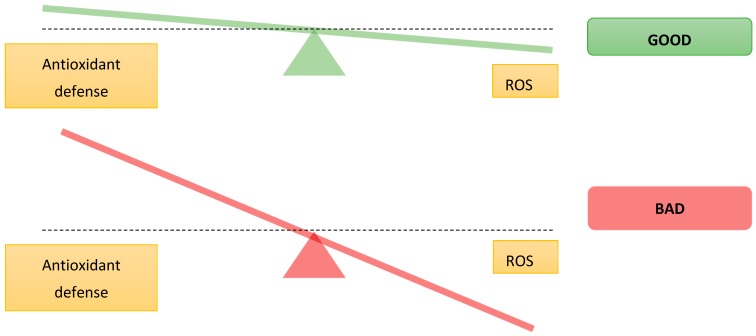
Difference between the normal and the disease state of oxidative stress balance adapted from Poljsak et al. [29].

It is well known that the production of ROS increases with age, while some of the endogenous defense mechanisms can decrease. If the balance between ROS and antioxidants is disturbed, the excessive amounts of ROS will damage cells by protein oxidation, DNA/RNA strand breakage, lipid peroxidation or the formation of advanced glycosylation end-products. These changes in the body with increased age lead to an aging phenotype related to neurodegenerative disease. However, it is important to remember that under normal conditions, a balance slightly on the pro-oxidant side is optimal for essential cell processes, such as cell signaling and redox regulation (Figure 2). Therefore, the antioxidant defense system should minimize the levels of harmful ROS, while enabling enough ROS to remain in the cell [29].

**Figure 3:**
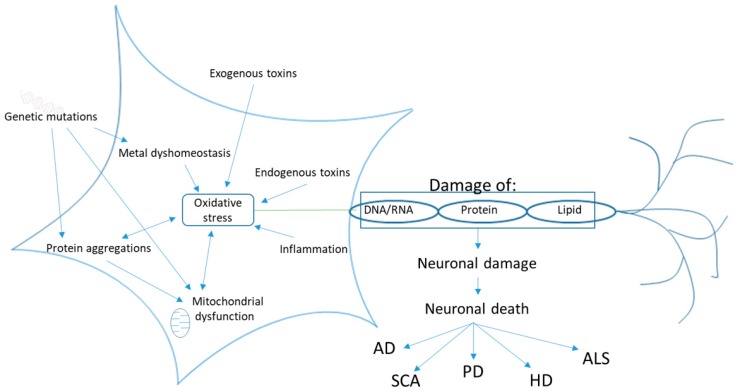
Causes of oxidative stress in neurodegeneration, AD—Alzheimer’s disease, SCA—Spinocerebellar Ataxia, PD—Parkinsons disease, HD—Huntington’s disease, ALS—Amyotrophic lateral sclerosis, adapted from [32,33].

Redox dysregulation can be caused by the ineffectiveness of the endogenous antioxidant system to handle an increase in the production of free radicals (e.g., disease, mitochondrial dysfunction, exposure to environmental factors) or because of a decreased effectiveness of the endogenous antioxidant system itself. These additional stress can cause damage to biological molecules that lead to rapid cell death, resulting in neurodegeneration, either by functional loss (ataxia) or sensory dysfunction (dementia) [30,31]. In addition, inflammation, protein aggregations (e.g., amyloid in AD), and excessive presence of metal ions such as iron (Fe^2+^) and copper (Cu^2+^) can cause oxidative stress (Figure 3).

### 2.4. Antioxidants to Counteract Oxidative Stress

**Figure 4:**
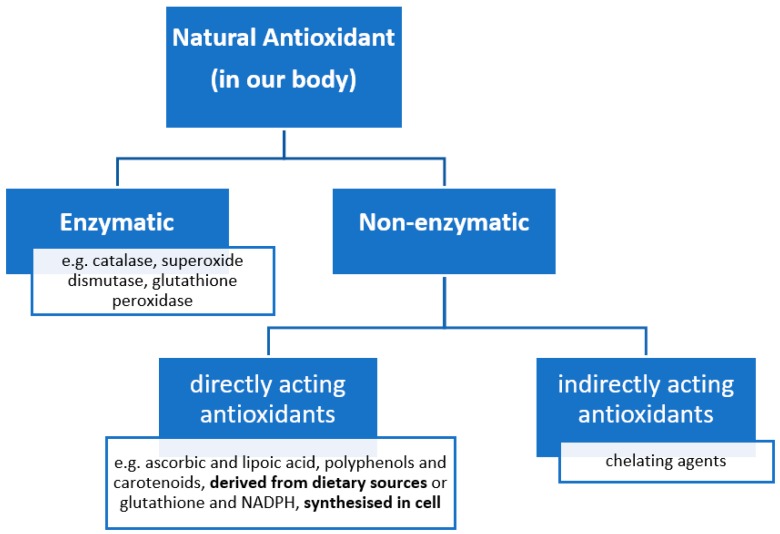
General natural Antioxidants categorized with examples, adapted from [16,34,35].

Generally, the human body has its own in-built antioxidant system (Figure 4), which ought to keep a balance between the production of ROS and the antioxidant defense system in our body. Keeping this biological equilibrium is especially important for neurons. Due to their very high oxygen consumption, long lifetime, and the additional formation of reactive nitrogen species and the prominent role of nitric oxide in the signaling processes, neuronal cells are even more susceptible to oxidative stress [16,30]. The natural human antioxidant system in our body can be divided in two groups, enzymatic and non-enzymatic [16] (Figure 4).

When the endogenous antioxidant system is not functioning well enough, an increased amount of external supply of antioxidants (exogenous antioxidants) could reduce the cumulative effect of oxidative stress [34], and hence prevent oxidative cell damage and neuron loss. Sources of available antioxidants can be either synthetic (chemically synthesized) or naturally occurring. A large variety of synthetic antioxidant compounds has previously been discussed by Augustyniak et al. [35] and Carocho and Ferreira [36]. However, these synthetic antioxidants are sometimes associated with a negative reputation, e.g., toxicity [36]. For this reason, in most antioxidant studies nowadays, compounds from natural sources are employed. One source of natural antioxidants is the human diet; plant products, especially, provide a good source of plant secondary metabolites, many of which have previously demonstrated positive antioxidant activities in vitro.

### 2.5. Natural Product Drug Discovery

In drug discovery pipelines, many plant natural products (single compounds isolated from natural sources) and/or plant extracts (containing various secondary metabolites) have shown antioxidant activity. Plant metabolites have provided inspiration for medicinal chemists and a platform for drug development processes for a very long time [37]. In the last two decades the use of plant metabolites in drug discovery has decreased, mainly due to the technical barriers for screening natural products in high-throughput assays for specific molecular targets, issues associated with the synthesis of natural compounds [38], advances in metagenomics, and the emergence of combinational chemistry [39]. However, more recently there has been a re-emergence of intense interest in plant metabolites. This is due to the advancement in improved fractionation and advanced NMR techniques for structural elucidation. This also includes profiling and isolation techniques such as HPLC-MS/MS, high-resolution Fourier-transform mass spectrometry and photo-diode arrays for metabolomics [38].

Biological activity detected in plants or plant extracts is mostly caused by plant secondary metabolites, which occur in plants at the higher level of structural diversity and number. They can fulfil a variety of different ecological roles in the plant, such as defense against herbivores, fungi, bacteria and viruses, as well as helping them compete for water, light and nutrients. In addition, they can also act as signaling compounds to attract pollinating and seed-dispersing animals, help to protect from external stress, such as UV light or physical harm, or have selected physiological functions [40]. According to Wink, secondary metabolites can be subdivided into two main categories, nitrogen-containing compounds and those without (Table 1). Examples of structures, pathways of secondary metabolite formation, and further details on each of the groups are provided by Wink [40].

Although some secondary plant metabolites are thought to have disease-protecting properties in vivo, they are non-essential nutrients for humans. Phytochemicals from plants were shown to exhibit different biological activities, such as anti-inflammatory [41,42,43,44], anti-microbial [45,46], anti-carcinogenic [46,47] and anti-diabetic properties [48,49]. Some of these are due to the regulation of several cellular molecular pathways [50]. Because of these varying properties, they are believed to have beneficial value for human health. Hence, the in vitro and in vivo effects of phytochemicals have shown intense interest as testified in the literature, including their ability to treat and prevent neurodegeneration [28,45,48,51].

## 3. In Vitro and In Vivo Antioxidant Activity of Plant Natural Products and Extracts

**Figure 5:**
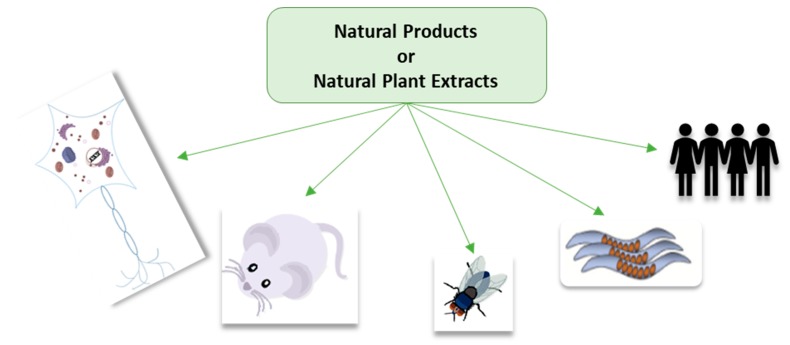
In vitro and in vivo models of neurodegenerative disease discussed in this review.

To study neurodegenerative disease, and the potential use of natural plant products or plant extracts for their prevention or treatment, different model organisms, such as rodents (mice, rats), fruit fly (*Drosophila melanogaster*), zebrafish (*Danio rerio*) and nematodes (*Caenorhabditis elegans*), and unicellular organisms, such as yeast (*Saccharomyces cerevisiae*), have been studied [52]. Other options available for the understanding of neurodegeneration and the effect of treatment are cell-based studies. Recent developments in stem cell research have made it possible to generate induced pluripotent stem cells, which have the capacity to self-renew by dividing and to develop into all cells of the adult body from differentiated patient-derived cells in the laboratory [53]. Advancing this research even further has led to the creation of 3D models using human stem cells [52,54,55]. Examples of in vitro and in vivo studies using single natural product from plants as well as plant extracts are presented in Figure 5.

### 3.1. In Vitro Cell-Based Research

When undertaking research on the effect of natural products in the field of neurodegeneration the best option for cell-based research is the use of primary neurons. Primary mammalian neurons can be derived from embryonic central nervous system tissue. However, the limitation of these is that once terminally differentiated into neurons, these cells will no longer propagate, and so the number of cells available for experiments is limited [56,57].

To overcome this issue, secondary cell lines, derived from neuronal tumors that have become immortalized can be used. The advantages of this are: (i) unlimited number of cells which grow easily in cell culture conditions; (ii) less variability; (iii) less challenging preparation and cell culture; (iv) approval from ethics committees is easier; and (v) facile transfection. There are cell lines currently being used for the search of potential treatment options from natural antioxidants, and these include neuroblastoma cell line SH-SY5Y, the human neuronally committed teratocarcinoma cell line NT2 or NTera, as well as the rat cell line PC12 derived from pheochromocytoma of the adrenal medulla and the HT22 mouse hippocampal neuronal cell line.

However, there are also disadvantages to using these neuron-like cell lines, such as more physiological differences to mature neurons, which make them less likely to show the same properties as matured neurons in vivo [57]. Depending on the field of interest, the right cell line/type needs to be chosen. Following all varieties of cell-related in vitro studies is considered to demonstrate the use of natural antioxidants in the field of neurodegeneration studies, using either whole plant extracts or single compounds found in plants.

In PC12 cells, Cheon et al. demonstrated the potential use of Korean mountain ash (*Sorbus alnifolia*) to protect dopaminergic neurons in the MPP^+^ (1-methyl-4-phenylpyridinium) induced model of PD. In this study, the MPP^+^-mediated oxidative damage led to neurotoxicity. The viability of neurons was improved by pre-treatment with the methanol extract of *Sorbus alnifolia* for 2 h [58]. The same group also studied the effect of the extract in MPP^+^ *C. elegans* models of PD, which are detailed later. Previous chemical analysis undertaken on extracts of *S. alniolia* had shown the presence of the following phenolic compounds: protocatechuic acid, p-coumaric acid, caffeic acid, quercetin and most of all chlorogenic acid, with a total phenolic content (Folin–Ciocalteu method) of 76.6 mg gallic acid equivalence (GAE)/g. The same study also showed the lack of toxicity from the extract (IC_50_ > 500 µg/mL) on RAW 264.7 and HS-68 macrophage cells [59].

Extracts from *Allium cepa* (onion) have also received a lot of attention in recent years. In a paper by Lee et al. in 2016, *Allium cepa* extracts were found to protect primary cortical neuronal cells derived from mouse embryos from oxidative stress mediated by extracellular signal-regulated kinase (ERK)1/2 phosphorylation and mitogen-activated protein kinase (p38MAPK) dephosphorylation and inhibition of protein kinase C (PKC-ε) [60]. In the same study, they verified that Allium cepa’s main flavonoid component quercetin showed the same effect. Furthermore, the ability of the latter extracts to protect: (i) pUC19 plasmid DNA from oxidative stress induced by Fenton’s reagent (ii) leukocyte DNA from H_2_O_2_-induced damage ( via comet assay) were verified by Fredotović et al. [61]. Of note several researchers had previously established the positive properties of onion extracts/quercetin in vivo [62,63,64].

**Figure 6:**
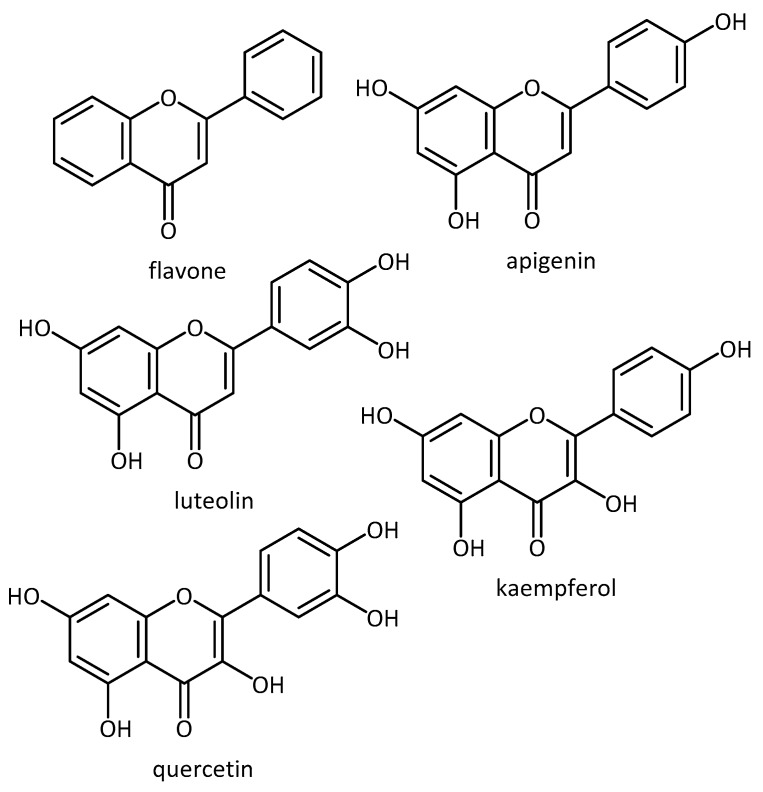
Structure of antioxidant flavonoids.

Furthermore, the use of polyphenols displaying antioxidant properties has been widely studied and has shown promising effects in different diseased models. For example, Pate et al. [65] showed the potential of five flavonoids (flavone, apigenin, luteolin, kaempferol and quercetin) to attenuate Aβ oligomer-induced neuronal responses associated with AD (Figure 6).

In that study, undifferentiated SH-SY5Y cells were induced with Aβ_1–42_ oligomers, leading to increased cellular ROS and caspase activity. The tested flavonoids altered oligomer size distribution and conformation. In addition, they were able to attenuate the oligomer induced intracellular ROS and caspases activation (only for luteolin and quercetin, Figure 6) [65]. In a different paper, both luteolin and apigenin was shown to induce Nrf2-mediated gene expression, thought to be responsible for their anti-inflammatory effects [66]. The general in vitro and in vivo antioxidant properties of flavonoids have previously been reviewed by Procházková et al. [67].

Other studies have looked into the properties of natural antioxidants that possess more than just antioxidant activity in vitro. [68]. For example, from a screened library of 84 antioxidants, 7 phenolics were selected which, apart from their antioxidant activity [67], also showed inhibition of kinase activity on leucine-rich repeat kinase-2 (LRRK2) substrates. Mutations in LRRK2 are known to cause familial PD; for example, the substitution of glycine for serine at amino acid position 2019 (G2019S mutation) is associated with increased kinase activity. Antioxidants that show inhibition of kinase LRKK2 substrates could turn out to be multi-target drugs for the treatment of PD.

**Figure 7:**
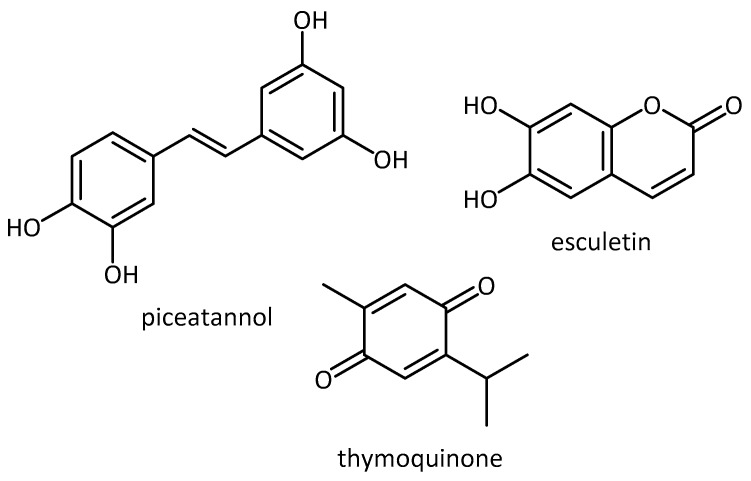
Phenolics from plants.

G2019S mutation-transfected SK-N-SH and cortical neurons, for example, showed increased viability when treated with piceatannol (phenolic found in grapes, passion fruit, white tea and Japanese knot [69]), thymoquinone (quinone active metabolite in *Nigella sativa* (black cumin [70]) and esculetin (coumarin found in bark of *Cortex fraxini* (traditional Chinese medicinal herb) [71]) [68] Figure 7. Compounds with both antioxidant and kinase inhibitory properties provide an added advantage when compared with single-target compounds, since they can hit two or more targets at the same time.

**Figure 8:**
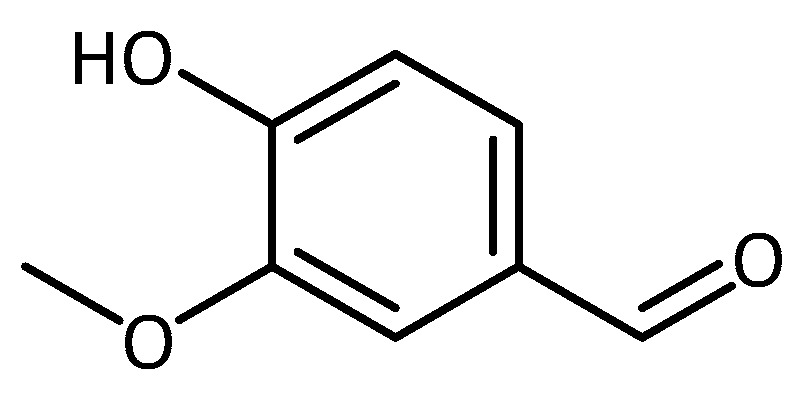
Structure of vanillin.

Vanillin (4-hydroxy-3methoxybenzaldehyde, Figure 8), a phenolic compound found in the beans and pods of tropical vanilla orchids (*V. planifolia, V.tahitensis and V. pompon*) currently used as food or beverage flavoring or in cosmetics and household products, has been shown to inhibit peroxynitrite-mediated reactions [72]. 

Although not exhibiting high DPPH scavenging activity, it does show activity in the ABTS●^+^-scavenging and the ORAC assays [73], and it protects plasmid DNA from AAPH-induced DNA strand breakage [74]. In an unrelated neurodegeneration study, vanillin was also found to be an anti-inflammatory agent [75]. Considering these positive characteristics, Dhanalakshmi et al. [76] tested Vanillin’s potential to protect SH-SY5Y cells from rotenone-induced PD-like neurotoxicity. Undifferentiated cells were pre-treated with vanillin (2h) and then co-treated with rotenone (24 h). Vanillin protected cells from rotenone-induced cell death, ROS generation, and changes in membrane potential, as well as apoptotic changes in cellular morphology and protein expression [76].

### 3.2. In Vivo Drosophila Models

Much research has been conducted on natural products displaying antioxidant properties and neurodegeneration on in vivo *Drosophila* models. Drosophila have several advantages over mammalian models; for example, their small size and low maintenance costs, easy genetic manipulation, short life cycle, large number of progenies, exhibition of complex behaviors, and less stringent ethical concerns. A review by McGurk et al. [77] summarizes the use of Drosophila in neurodegenerative disease research.

**Figure 9:**
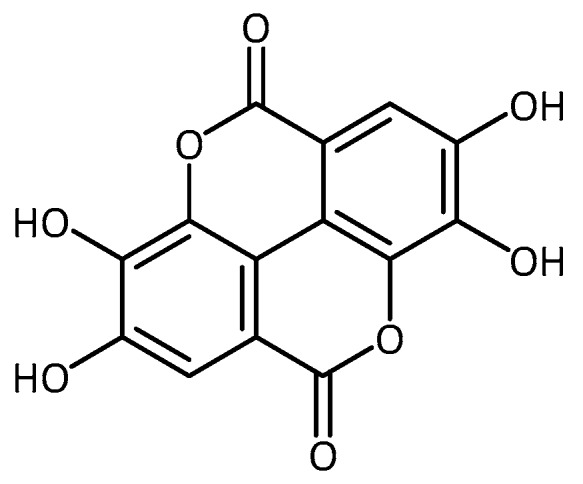
Structure of ellagic acid.

An interesting paper by Jahromi et al. [78] utilized drosophila PD models (missense A30P mutations and A53T α-synuclein) to test the biological activity of swallowroot (*Decalepis hamiltonii*). Transgenic flies fed with the aqueous *Decalepis hamiltonii* extracts (0.1 and 0.5%) showed significantly improved climbing ability as well as circadian rhythm of locomotor activity. These motor improvements were associated with reduced ROS and lipid peroxidation, and with increases in CAT and SOD activity. Similar positive results were obtained with the deteriorated phenotype after treatment with paraquat. However, an improvement in climbing ability of the transgenic flies, as well as protection from mortality, was observed for both models at 0.1% and 0.5% treatment concentrations. Preliminary chemical analysis of *Decalepis hamiltonii* revealed the presence of ellagic acid (Figure 9), a well-known antioxidant plant secondary metabolite. Both the extracts and ellagic acid on its own showed radical scavenging activity together with metal-chelating activity in vitro [79,80]. Ellagic acid also exhibits cyto-protective activity in EAT cells following stress induction with hexachlorocyclohexane (HCH), cumene hydroperoxide (CHP) and carbon tetrachloride (CCl_4_). In addition, xenobiotic-induced ROS production and lipid peroxidation was inhibited, together with the prevention of GSH depletion [80].

Another recent study [81] looked into the activity of Peacocks tail (brown algae; *Padina pavonica*) and barbary fig (*Opuntia ficus-indica*), which had previously been shown to exhibit very strong antioxidant activity [82,83], and had provided ameliorating effects in neurodegenerative disease models in Drosophila (AD, PD) and yeast (AD). Both extracts were able to improve the survival and mobility in AD models. The observed improvements were found for two different disease models, whereas *Padina pavonica* extracts improved a pan-neuronal expression of a double dose of Aβ42 (late-onset AD model), *Opuntia ficus-indica* showed positive effects in a model of early-onset AD (flies expressing the Arctic Aβ42). Furthermore, both extracts improved survival of α-syn^A53T^ expressing PD model and attenuated Aβ42 and α-syn oligomer toxicity while demonstrating anti-amyloidogenic potential against both proteins in vitro [81].

While Angeles et al. [68] found increased viability when treating G2019s transfected SK-N-SH and cortical neurons with piceatannol, thymoquinone and esculetin (see above), they also tested these three compounds in G2019S-expressing transgenic flies. Their results showed that these kinase inhibitors also provide protection from loss of dopaminergic neurons, suggesting amelioration of LRRK2 induced toxicity. In addition, the flies’ motor abilities (climbing), impaired in G2019S animals, were improved with piceatannlo and thymoquinone treatment. Both esculetin and thymoquinine also reversed the decrease in life expectancy induced by the mutation.

### 3.3. In Vivo C. elegans Models

In addition to Drosophila, the nematode *C. elegans* is a well-established model for neurodegenerative disease research. A review by Alexander et al. provides a good overview on the different *C. elegans* models available for AD, PD, ALS and others with details on the advantages and limitations of these models [84].

**Figure 10:**
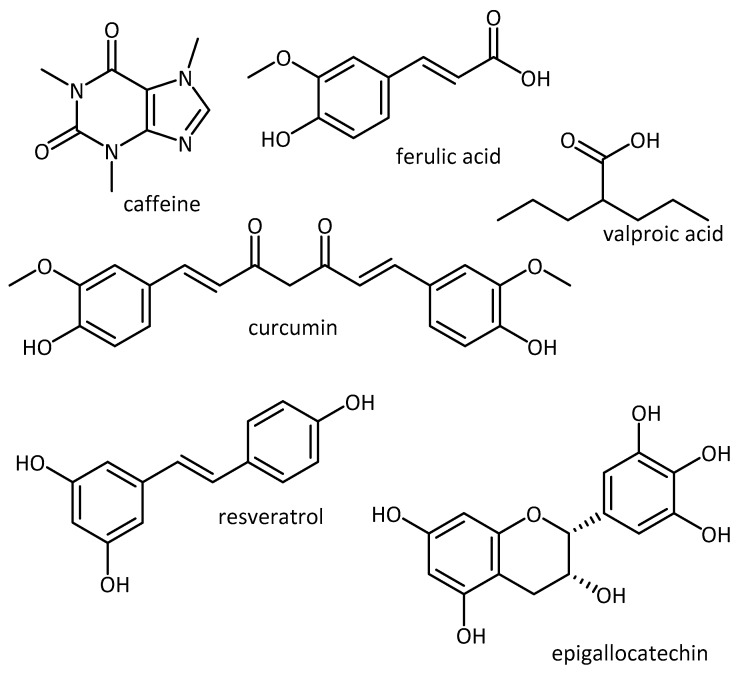
Natural products used in the study of aging-associated neurodegenerative diseases.

A further review on “Using *C. elegans* to discover therapeutic compounds for aging-associated neurodegenerative diseases” by Chen et al. [85] goes into further depth in using *C. elegans* models in the search for treatments for neurodegeneration and lists compounds which have shown beneficial effects, including secondary metabolites such as caffeine, curcumin, epigallocatechin, ferulic acid, resveratrol and valproic acid (Figure 10).

Both natural products isolated from plants and plant extracts containing a vast number of active ingredients have been used and have shown positive effects in various neurodegenerative disease models. Methanol extracts of tea seed pomace from *Camellia tenuifolia* (oil-tea tree), for example, decreased intracellular ROS and prolonged lifespan and survival nder oxidative stress in Bristol N2 (wild type). In an AD model, the extract was able to reduce β-amyloid (Aβ) toxicity in transgenic *C. elegans* expressing human Aβ (strain CL4167). Its radical scavenging activity and phenolic content were verified using the DPPH and the Folin-Ciocalteu method, respectively. Fractionation demonstrated that one fraction was particularly active. Semi-preparative HPLC analysis was used to determine the constituents present in this fraction as kaempferol derivatives kaempferol 3-*O*-(2″-glucopyranosyl)- rutinoside and kaempferol 3-*O*-(2″-xylopyranosyl)-rutinoside, which, when tested in the oxidative stress-induced survival assay, showed a significant increase of protection [86].

In a PD model induced by the treatment of *C. elegans* with MPP^+^, the extract of Korean mountain ash (*Sorbus alnifolia*) restored viability of the worms after 30 min pre-treatment prior to MPP^+^ addition. Using the transgenic strain BZ555 ((egIs1, Pdat-1::GFP) expressing GFP in the 8 dopaminergic (DAergic) neuron of hermaphrodite *C. elegans*, observations also showed that the extract protected DAergic neurons from MPP^+^ (environmental) induced neuronal loss [58]. The same was true for the genetic PD model UA57 (Pdat-1::GFP and Pdat-1::CAT-2), where the overexpression of tyrosine hydroxylase led to DAergic neurodegeneration. Extract concentration of 250 µg/mL showed the best neuroprotection in both models. The extracts, however, failed to prevent aggregation of α-synuclein in a transgenic strain (NL5901). Further studies into the phenotypical induced behavioral dysfunction induced by MPP^+^ (basal slowing response) demonstrated the extracts’ activity in reducing the phenotypical dysfunction. In addition, the extract increases the overall life span in N2 (wild type) animals [58].

In non-mammalian model organisms, the use of both pure natural products and extracts is common. There is also increasing evidence that using either single compounds or a mixture of compounds (mix of individual compounds or extracts) acting on several molecular targets or pathways could be a more effective therapeutic strategy than using natural products. Drug trials like this can be more rapidly undertaken in *C. elegans* or *Drosophila* models compared to rodents. Advanced molecular methods such as RNAi and CRISPR have made it easier to create models of neurodegenerative disease more rapidly and accurately [85]. Single copies of mutant genes found in patients can now be delivered precisely to appropriate locations in the model genome, without long-lasting gene editing or crossing. This might lead to faster and better understanding of disease mechanisms and potential ways of treatment.

In our own research, using antioxidant rapeseed (*Brassica napus*) pomace/cake extracts [87,88], we showed neuroprotective properties in a *C. elegans* model of SCA3 or Machado-Joseph disease. Further investigation also found positive effects in different PD *C. elegans* models. Currently, we are studying the mechanism of activation of the antioxidant pathways after treatment with RSP extract (unpublished).

### 3.4. In Vivo Rodent Models

Cellular and invertebrate models can yield much useful information. However, due to their simplistic nature, detailed information/mechanisms from these models can be limited when it comes to translational research [89]. In the field of neurodegenerative research, rodent models are still important for pre-clinical models, which we cannot evade when searching treatments for neurological disorders using either natural or synthetic entities. Several reviews have reported on the background of the different rodent models available for modeling neurodegenerative diseases, including both genetic and potential pharmacologic models [90,91,92]. Rodent models, just like non-mammalian models, come with several advantages and disadvantages. Some of the advantages are the closer genetic similarity to humans and their higher complexity compared to, e.g., *C. elegans* and *Drosophila melanogaster* or in vitro cell models [93]. Although translation of research from rodent models to human trials on disease modifying therapies remains an issue [90], it is worth mentioning that the number of papers (between 30–40% from 1975 to 2015) using rodent models still outweighs the number of any other publications in neuroscience, as presented by Keifer and Summers [93]. 

**Figure 11:**
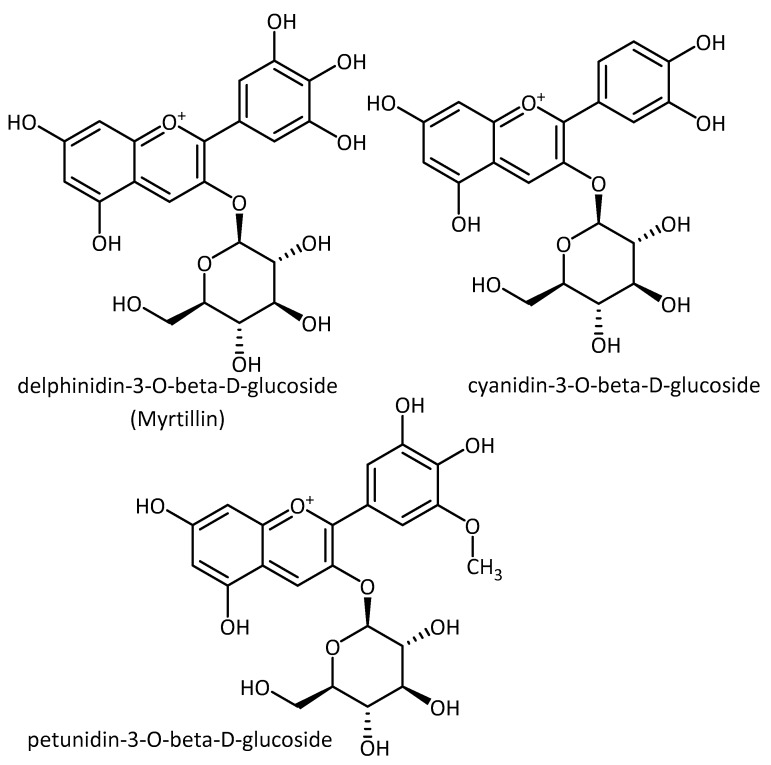
Structure of anthocyanins.

In a recent study by Ali et al. [94], an APP/PS1 (AD) mouse model was employed, containing human transgenes for both amyloid precursor protein (APP) and the L166P mutation of presenilin (PS-1) [95]. In this model, anthocyanins (Figure 11) extracted from Korean black soybeans (methanol 95% extract, purified in a XAD-7 column) was shown to regulate the PI3K/Akt/GSK3 pathway, activated the downstream endogenous anti-oxidant Nrf2 transcription factor and its target genes HO-1 and GCLM. By doing so, the amyloid β oligomer (AβO)-induced elevation of ROS was reduced and neurodegeneration via a PI3K/Akt/Nrf2-dependent pathway was prevented. In this study, anthocyanins improve memory-related pre- and postsynaptic markers and cognitive functions in APP/PS1 mice. Similar results were also found in their AβO-exposed HT22 mouse hippocampal neuronal cells [94]. Major anthocyanins in black soybean have previously been identified as delphinidin-3-*O*-β-d-glucoside, cyanidin-3-*O*-β-d-glucoside and petunidin-3-*O*-β-d-glucoside [96].

In vitro and in vivo data collected in the study by Ali et al. [94] showed that anthocyanins and secondary metabolites act as potential antioxidants by activating antioxidant pathways, and hence could be beneficial for the prevention of age-related neurological disorders, e.g., AD.

**Figure 12:**
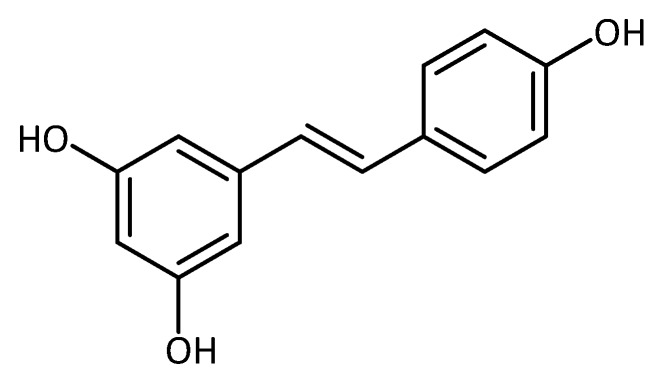
Structure of resveratrol.

Another well-researched secondary metabolite, known to have antioxidant activity in vitro [97], is resveratrol (Figure 12). Several studies in mice have suggested its use for the treatment or prevention of neurodegenerative disease. For example, in a SCA-3 (Machado-Joseph disease, MJD) mouse model, resveratrol activates the histone deacetylase enzyme SIRT1 pathway, showing improvement in motor behavior when treating animals at a post-symptomatic stage of disease development. In that study, the resveratrol data were compared to caloric restriction, which also decreases motor deficits via activation of SIRT1 [98]. The effect of resveratrol has previously been recorded in different neurodegenerative disease models (AD, HD, ALS, PD), both in vitro and in vivo [99].

**Figure 13:**
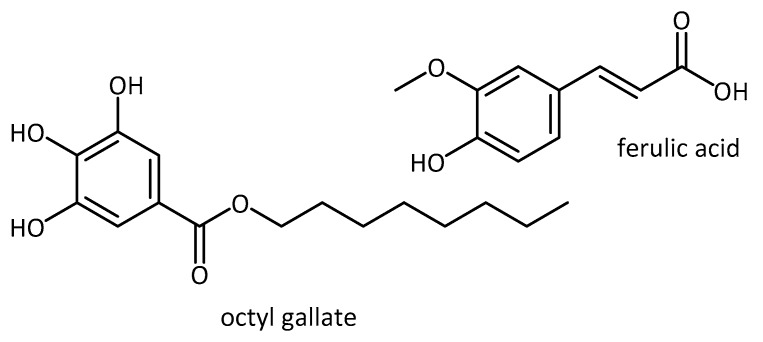
Natural products used in combination treatment.

Single compounds do not always show the same positive effect when moving from in vitro to in vivo environments or to clinical trials. This could be due to the bioavailability of natural products in an in vivo setting. A newer trend in the search to treat or prevent neurodegenerative disorders is combination therapy. New assumptions are that a combination of different compounds, as seen in many extracts, or a mixture of natural products, could lead to better results. Mori et al. [100], for example, used a combination treatment of octyl gallate (antioxidant activity [101]) and ferulic acid (neuroprotective [102] and in vitro antioxidant activity [103] Figure 13) in a mouse model of AD (PSAPP transgenic mouse model of cerebral amyloidosis). 

The results were compared to single compound treatment, and the combination treatment showed significantly better results than the single compounds. The combination treatment led to improved cognitive functions, reduced level of β-amyloid deposits, and amyloid β-protein abundance, as well as attenuated neuroinflammation, oxidative stress and synaptotoxicity [100]. Ferulic acid has not only shown success in mouse, but also in rat models of neurodegeneration (see below).

**Figure 14:**
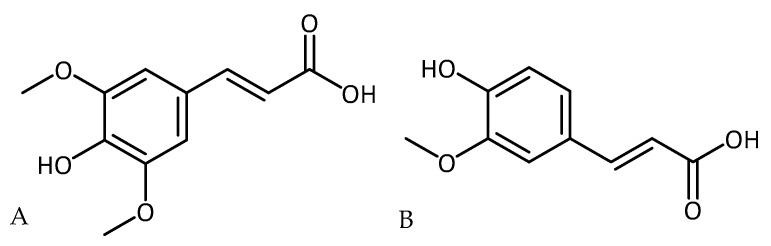
Structure of (**A**) sinapic acid and (**B**) ferulic acid.

In a rotenone-induced rat model of PD, ferulic acid (Figure 14), a common phenolic acid in many plants, rescued dopamine neurons in the substantia nigra pars compacta area and nerve terminals in the striatum from the rotenone insult. In addition, ferulic acid restored antioxidant enzymes (SOD and CAT), prevented glutathione depletion levels, and inhibited lipid peroxidation (MDA level). Inflammatory mediators and pro-inflammatory cytokines were reduced. The observed positive effects are thought to be mediated by the phenolic acid’s antioxidant and anti-inflammatory properties [104].

In comparison, in a 6-OHDA-induced model of PD (unilateral intrastriatal), Wistar rats were pre-treated with sinapic acid (Figure 14), showed improved turning behavior and the prevention of neuron loss in the substantia nigra pars compacta so common for PD. In addition, sinapic acid reduced the levels of MDA and nitrite and lowered iron reactivity. These results led to the assumption that neuroprotection is at least partially due to antioxidant activity [105] and sinapic acid’s potential to lower iron levels in vivo [106].

In general, it would appear that the number of studies related to plant extracts decreases when moving from in vivo models such as *C. elegans* and *Drosophila* to mammalian models such as mice and rats. In Kaur et al., rice bran extracts were used to study their beneficial effect in 3-nitropropionic acid induced experimental HD rats. In that study, both the hexane and ethanol extracts of rice bran were able to attenuate the 3-nitropropionic acid induced behavioural, biochemical, neuro-inflammatory and neurochemical changes, suggesting the potential use of rice bran as adjuvant or prophylactic therapy. 

**Figure 15:**
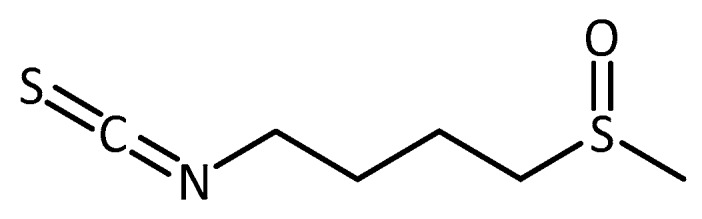
Structure of sulforaphane.

More often, pure natural products are tested, for example sulforaphane, an isothiocyanate (Figure 15) known to originate from the breakdown of glucoraphanin, a glucosinolate present in many cruciferous vegetables. In an AD mouse model (induced by the combined administration of d-galactose and aluminum), sulforaphane was able to improve neurobehavioral deficits and protect mice brains from Aβ deposits and peroxidation [107]. Similar positive results for sulforaphane were found in other rodent models of AD [108], HD [109] and PD [110]. Sulforaphane is a well-researched Nrf2 transcription factor activator, and hence it has received a lot of attention in related non-animal studies [111,112,113].

Another isothiocyanate 4-(α-L-Rhamnosyloxy)-benzyl, present in *Moringa oleifera* (drumstick tree) seeds, after breakdown of glucomoringin by myrosinase, was able to delay the disease phenotype of a SOD1^G93A^ rat transgenic model of ALS. Again, natural products were used instead of using the plant material itself or extracts obtained from it.

Below, Table 2 highlights a few of the natural antioxidants that have shown beneficial effects in in vitro and in vivo animal models, including several examples discussed above, as well as additional recent studies.

## 4. Clinical Trials: Positive vs. Negative Outcomes

The interest in using natural products in clinical trials has recently been on the increase. In 2017, the National Centre for Complementary and Integrative Health (NCCIH) introduced new funding opportunities for natural product clinical trials through a webinar [122].

**Figure 16:**
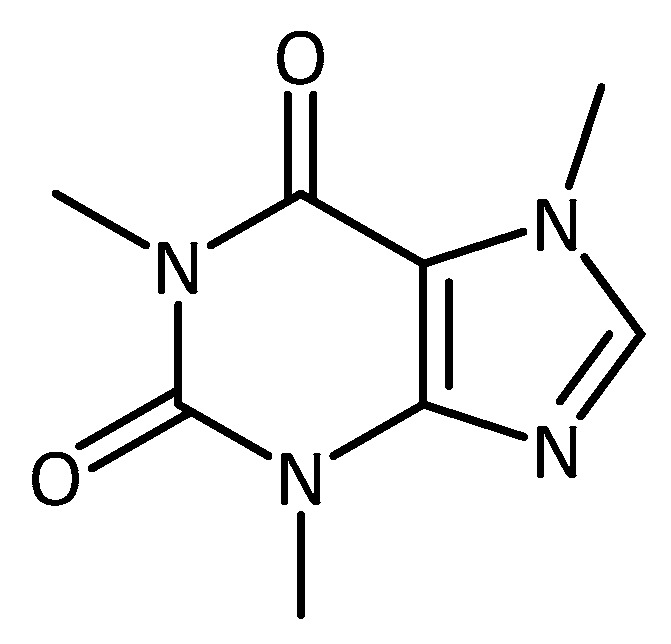
Structure of caffeine.

Caffeine (Figure 16) is a well-known secondary plant metabolite with good antioxidant activity and is under constant research for its health benefits. Caffeine has already shown promising results in both in vitro and in vivo animal models of neurodegenerative disease. In addition, different clinical trials have been undertaken using caffeine as a therapy for PD. Initial studies (2010/2011, Phase 2, estimated enrolment 28 participants) on the tolerability of caffeine, as well as secondary outcome measures such as Epworth Sleepiness Scale, Unified PD rating scale, timed up and go, and others were analyzed (NCT01190735). No record of the results from this study are available; therefore, the outcome remains unclear. Under the same investigator (Ronald B. Postuma, McGill University Health Center) a further Phase III study took place using caffeine as well as a placebo control with 119 participants, randomized and double blinded (NCT01738178). The results from that study concluded that caffeine did not show clinically relevant improvements in the motor manifestations of PD. Links between caffeine and a lower risk of developing PD do not seem to be explained by symptomatic effects [123]. Unfortunately, no molecular measurements of protein aggregations or changes in antioxidant levels in the patients were undertaken.

**Figure 17:**
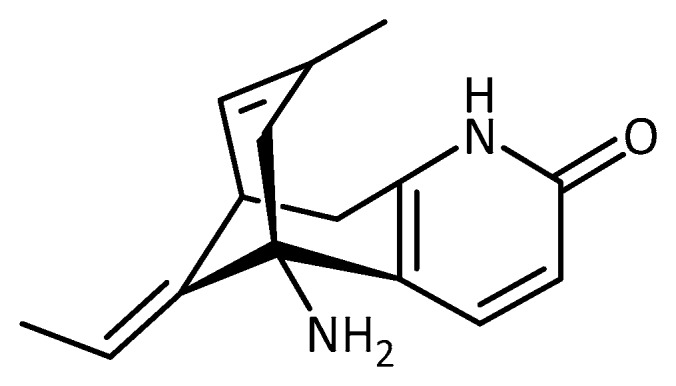
Structure of huperzine A.

Another natural product widely referred to its antioxidant activity is huperzine A (Figure 17), a cholinesterase inhibitor derived from the Chinese herb *Huperzia serrata*. In addition to its acetylcholinesterase inhibition activity, huperzine A has been shown to reduce the amounts of soluble and insoluble β amyloid levels and levels of amyloid plaques in AD mice. Mice on a high-iron diet, however, showed no reduction [124]. This and other “non-cholinergic” effects seen by the treatment with huperzine A are documented by Qian and Ke [125]. In a phase II trial (2004–2007, NCT00083590) investigating huperzine A, with 210 patients (177 completions) diagnosed with probable AD, with doses of 200 µg twice daily (BID- *bis in die*, Latin), no significant changes in the AD Assessment Scale cognitive subscale (ADAS-Cog) were seen after 16 weeks. However, secondary analysis at 400 µg concentration of BID showed improvements at 11 and 16 weeks, suggesting higher doses might improve cognitive functions [126]. Another review by Yang et al. took a further 20 randomized clinical trials into review, where the potential use of huperzine A in the improvement of cognitive function, daily living activity and global clinical assessment for AD patients was analyzed. However, the conclusion also suggested caution with the results, as some of the studies showed poor methodological quality and the suggestion was made for more rigorous clinical trials to support clinical use of huperizine A. An issue with all the trials included in this review (n = 20 published between 1995 and 2012) is the fact that none of them used concentrations higher than 300 µg BID. Nevertheless, as shown in the previous study, only concentrations above 200 µg BID showed promising results. The question is why so many clinical trials have been undertaken at concentrations below the effective concentration.

Due to a lack of significant clinical improvements using single antioxidant compounds, clinical trials have started to move away from single compounds and are starting to look into the use of plant extracts. For example, one clinical trial, started in 2014 (ClinicalTrials.gov Identifier: NCT02033941), studied the effect of Meganatural-Az Grapeseed Extract in a randomized placebo-controlled phase II trial. The results from this study were expected in September 2018, but no publications on the outcomes were found by the time of submission of this review. The aims of this study are to determine the safety and pharmacokinetics of grapeseed extracts in participants with AD. In addition, clinical and biomarker indices will be assessed to determine therapeutic efficacy. However, the low number of participants (20) might make it difficult to see significant results. On the other hand, it will hopefully be able to provide necessary human data to direct further studies using the extract with a higher number of subjects.

Miroddi et al. reviewed a total of eight clinical trials using two *Salvia* species, known as “common sage” and Spanish sage (*S. officinalis L. and S. lavandulaefolia L.*, respectively), to assess their pharmacological properties on memory and cognitive impairment in AD. All those trials were based on whole herbal extracts. Trials conducted on Salvinorina-A, an isolated compound from a different species of the *Salvia* genus, were not included. The conclusion from this review revealed several clinical trials on both *Salvia* species to show improved cognitive performance in healthy subjects and patients with dementia/cognitive impairment. No serious adverse effects were observed with treatment when compared to a placebo control. However, one issue highlighted in this review was the inconsistent preparation of herbal products and a lack of details on the products used [127]. Such a lack in quality control for the preparation of natural extracts is worrying and could explain why clinical trials are more often done on a single compound with a specific dose. When undertaking clinical trials on extracts, their production, chemical composition and the origin of the plant material must be clearly indicated. An extraction method able to produce a product for the market also needs to be considered.

## 5. *Ginkgo Biloba* Extract EGb 761^®^: A Plant Extract Story with Varying Clinical Trial Outcomes

**Figure 18:**
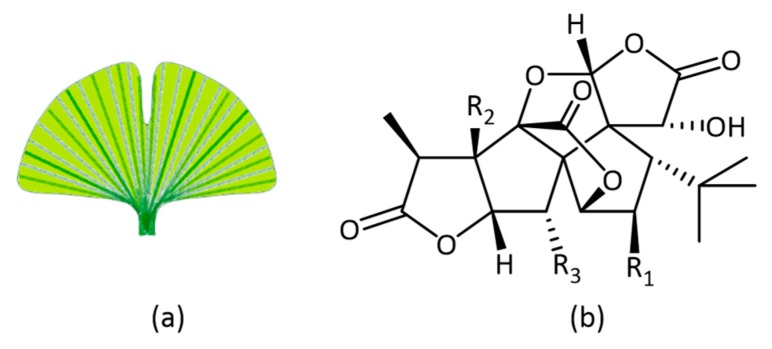
(**a**) Ginkgo leaf (**b**) ginkgolides.

The *Ginkgo biloba* (G*inkgoaceae*) tree, also described as a living fossil, has existed on earth for more than 200 million years [128,129]. *Ginkgo biloba* extract EGb 761^®^ is a standardized extract of *Ginkgo biloba* leaves, and has been widely studied in vitro and in vivo. The EGb 761 extract is well characterized and contains between 22–27% flavonol glycosides and terpene lactones (5–7%), including ginkgolides (Figure 18) and biloballides [130]. It is one of the herbal extracts that has made it onto the market as a dietary supplement, requiring no approval by the FDA. Several clinical studies had shown a positive effect in the treatment of dementia. On its way to market, the *Ginkgo biloba* extract passed successfully through all the stages of drug research, as described in further detail below. *Ginkgo biloba* has become one of the most widely studied medicinal plant products [129].

### 5.1. In Vitro Activity of Ginkgo Biloba

Very early research into the in vitro antioxidant activity of *Ginkgo biloba* extracts found the presence of significant amount of phenolics, ferric ion reducing antioxidant power, copper chelating ability [131], peroxyl radical scavenging activity [132] and radical scavenging activity [133].

In vitro studies with PC12 neuronal cells investigating Aβ(1–42)treatment (aggregated and soluble form) showed that *Ginkgo biloba* extracts have the potential to prevent Aβ-induced ROS production, cytotoxicity, glucose uptake and apoptosis in PC12 cells. In addition, the formation of Aβ-derived diffusible neurotoxic ligands was prevented. These neurotoxic ligands have been suggested to mediate the neurotoxic effect of Aβ [130].

### 5.2. In Vivo Activity of Ginko Biloba

In *C. elegans*, *Ginkgo biloba* extract EGb761 alleviates Aβ-induced pathological behavior, inhibits Aβ oligomerization and deposits (not by reducing oxidative stress), and attenuates the basal as well as the induced levels of H_2_O_2_-related reactive oxygen species in AD models of neurodegeneration [134,135].

In a mouse trial reported by Liu et al. in 2015, further in-depth research of *Ginkgo biloba* extracts was carried out to elucidate the anti-inflammatory and underlying molecular pathways in APP-transgenic mice. The results showed inhibition of neuroinflammation, reduction of cognitive deficit and synaptic impairment, and enhanced autophagy, as well as the prevention of Aβ-induced microglial inflammatory activation [136].

### 5.3. Clinical Trials of Ginko Biloba

Rainer et al. [137] conducted a meta-analysis on three clinical studies from Austria. Findings showed a delay in “activities of daily living (ADL)” deterioration and overall cost savings compared to cholinesterase inhibitor treatment options. This study, however, only looked at ADL and the costs involved, focusing on the delay in progression towards higher care requirements; neuropsychiatric symptoms were not considered. This was assessed in a placebo-controlled, double-blind randomized trial by Herrschaft et al., where patients treated with EGb 761^®^ showed increased performance in cognitive tests and improved neuropsychiatric symptoms, with fewer changes seen in the placebo group. Also, secondary outcome measurements, including quality of life, ability to cope with demands of everyday life and clinicians’ global judgement, were improved compared to placebo patients [138,139].

In a different phase III randomized controlled clinical trial (NCT00010803), a 120 mg BID dose was not able to prevent or delay the overall incidence rate of dementia or AD cases in participants with normal cognition/mild cognitive impairment [140].

A systematic review on the efficacy and adverse effects of *Ginkgo biloba* for cognitive impairment and dementia considered nine clinical trials (prior to 2014). Their findings showed that concentrations of 240 mg/day were able to stabilize or slow the decline of cognition, function, behaviour and global change in patients with cognitive impairments [141].

A new clinical study into the cognitive function of patients with mild to moderate AD using 3 different interventions (*Ginkgo biloba* dispersible tablets vs. Donepezil vs. *Ginkgo biloba* dispersible tablets and Donepezil) is targeting enrolment of about 240 participants, and study started in March 2018. This Phase II/Phase III study will use different primary outcome measures, such as Mini–Mental State Examination, ADAS-cog, Magnetic Resonance Imaging and others to determine the effect of the three treatment options on AD patients. By doing so, they will hopefully be able to shed more light onto the use of *Ginkgo biloba* extracts and its effect on AD patients (*NCT03090516*).

## 6. Conclusion/Future Perspectives

Although pure natural products or plant extracts displaying antioxidant activity have shown very good results in in vitro and in vivo animal models, their clinical outcomes in human patients are still inconclusive and demonstrate limited success. This could partially be due to the fact that in clinical trials, mostly single compounds are studied. In contrast, investigation of plant extracts containing a variety of secondary metabolites is more common in studies prior to clinical studies. The combination of different active ingredients in extracts can lead to additive or synergistic effects, giving better antioxidant/disease-modifying activity [142,143]. This may be one reason why, for example, the clinical trial with Meganatural-Az Grapeseed Extract examined the effect of whole extracts compared to single compounds, such as resveratrol found in grapeseed extracts, which had shown positive effects in some AD trials [144]. In general, clinical trial outcomes for phytochemicals have been highly variable, perhaps due to the way these trials are conducted. Clinical trials look at a wide variety of participants with different environmental and genetic background and even different disease symptoms and sometimes stages of disease. It might be worth taking a closer look, not at the general significances of the whole participant population, but at single individuals, or smaller groups of individuals, which do show significant improvement and determine why they might be responding to the treatment when others are not. Although this would be associated with extra cost in trial, it could lead to a better understanding of the potential use of antioxidants in certain groups of patients, either with a certain genetic or environmental background, which would also lead to better understanding of the neurological disorder. In general, most clinical trials on natural antioxidants (i.e., natural products or plant extracts) have only looked at behavioral or cognitive improvements in patients, very few trials were found that actually assessed molecular markers of the disease or oxidative stress specifically.

Multi-target drugs and drug cocktails (combination of single compounds or extracts) also need to be further investigated. This consideration contradicts the one drug one disease (or one target one disease) approach which is currently used for many different disease treatments. However, as neurodegenerative disease can be associated with multiple cellular dysfunctions caused by various external and internal factors, a multi-target drug approach might be a better way forward, especially due to the complex nature of these diseases [49,145]. Multi-target drugs have received more attention in recent years, as evidenced by the drug approval of Namzaric in 2014 [9], which is a combination treatment of memantine hydrochloride (extended-release) and donepezil hydrochloride, which are a NMDA receptor antagonist and an AChE inhibitor, respectively. The multidrug treatment option was further investigated in a recent study by Admasu et al. [146] in *C. elegans*, in which combinations of currently FDA-approved drugs led to significant healthspan and lifespan elongations. Natural antioxidants (i.e., from natural products or plant extracts) could also fulfil this multi-target drug profile. They have shown antioxidant activity, but have also presented other activities beneficial to neuronal health, such as metal chelating, anti-inflammatory, AChE inhibition or anti-protein aggregation activities. Another option is the use of FDA-approved AChE inhibitors such as donepezil, rivastigmine, or galantamine, or even less-active AChE inhibitors in conjunction with natural antioxidants. Drug mixtures with moderate activities acting on two or more targets might be more effective than a single highly effective but selective drug acting on a single target [147].

Apart from this, a better understanding of the mechanisms causing the disease are necessary. For most neurodegenerative diseases, the exact cause of the disease is still only partially understood, if not completely unclear. Some of them, e.g., PD, can be caused by environmental or genetic factors, while for others, such as HD, the cause—a genetic mutation in the huntingtin gene—is well known, but the exact disease mechanism/pathway is still unclear, as the behavior of the mutated protein has not been completely elucidated. Very often, the reasons why particularly neurons are affected by, for example, mutated proteins, is still unclear. Constant research into improved animal models for these diseases might help with both a better understanding of the neurological disorder and the search for potential drugs. A special collection of articles on “Neurodegeneration: from Models to Mechanisms to Therapies” from 2017 covers a few of the important issues in different neurodegenerative disease [52].

At a recent national ataxia foundation conference, the issue of pre-treatment versus treatment once the disease has developed was brought to attention. Several people agreed that pre-treatment might be a possible option. This, however, would make it necessary to find better biomarkers that would make it possible to determine people who might turn into patients later on. In this case, we might also find responders and non-responders. Non-responders would then have the chance to move into treatment options, which have proven successful once the disease showed initial symptoms. Natural product or plant extract antioxidants might be a great option for potential pre-symptom treatment, as they are most often not associated with side effects and are well accepted in the population. Long-term trial would need to verify this, of course.

In conclusion, plant natural products and extracts have shown several positive effects in in vitro, as well as in vivo, neurodegenrative disease models. However, translation to clinical trials is lacking in positive outcomes. Further reseach into the pathology and mechanisms of these neurodegenerative diseases might help to elucidate the lack of success of plant extratcs and natural products in human patients.

## Figures and Tables

**Table 1:** Secondary metabolite groupings.

Nitrogen-Containing	Without Nitrogen
Alkaloids	Terpenes (Mono-, Sesqui-, Di-, Tri-, Tetraterpenes)
Non-protein amino acids	Steroids, saponins
Amines	Flavonoids, tannins
Cyanogenic glycosides	Phenylpropanoids, lignin, coumarins, lignans
Glucosinolates	Polyacetylenes, fatty acids, waxes
Alkamides	Polyketides
Lectins, peptides, polypeptides	Carbohydrates, organic acids

**Table 2:** Pre-clinical effect of natural antioxidants in vitro and in vivo.

In Vitro/ In Vivo	Origin of antioxidant/s	Model system	Condition	Molecular Outcome
In vitro	Korean mountain ash (*Sorbus alnifolia*)	PC12 cells	PD	- restored MPP^+^-induced loss of viability [58]
	Onion (*Allium cepa)/* quercetin	primary cortical neurons derived from mouse embryos	Oxidative stress	- protection of cells mediated through ERK1/2 phosphorylation and p38MAPK dephosphorylation and inhibition of PKC-ε [60]
	Vanillin	SH-SY5Y	Neurodegeneration in general	- attenuated rotenone induced mitochondrial dysfunction, ROS generation, oxidative stress, and apoptosis [76]
	Flavonoids		AD	- flavonoids altered oligomer size distribution and conformation and were able to attenuate the oligomer induced intracellular ROS and caspases activation (only for luteolin and quercetin) [65]
	Pomegranate Juice Extracts	Primary human neurons	PD	- ameliorate MPTP-induced neurotoxicity [114]
	Caffeic Acid and Resveratrol	SK-N-SH-MJD78	MJD/SCA3	- decreased reactive oxygen species (ROS), mutant ataxin-3 and apoptosis and increased autophagy in pro-oxidant tert-butyl hydroperoxide (tBH)-treated cells [115]
	Piceatannol, thymoquinone, esculetin	SK-N-SH -G2019S	PD	- increased viability through multi-target approach of antioxidant and kinase inhibitory properties (LRRK2 model of PD) [68]
In vivo	Korean mountain ash (*Sorbus alnifolia*)	*C. elegans*	PD	- protection against chemically and genetically induced DAergic neurodegeneration, increased food-sensing functions and prolonged average lifespan [58]
	Tea Seed Pomace (*Camellia tenuifolia*)		AD, aging	- decreased intracellular reactive oxygen species, prolonged lifespan and reduced amyloid-β (Aβ) toxicity in transgenic *C. elegans* expressing human Aβ [86]
	Extract from red seaweed (*Chondrus crispus*)		PD	- decreased the accumulation of α-synulein and protection from 6-OHDA induced dopaminergic neurodegeneration, improved movement, potentially associated with up-regulation of the stress response genes, sod-3 and skn-1 [116]
	Betulin (e.g., from outer bark of birch trees)		PD	- decreased a-syn accumulation in the transgenic C. elegans model and reduction of 6-OHDA-induced dopaminergic neuron degeneration, improved food-sensing behavioral and reversed life-span decreases in a pharmacological C. elegans model [117]
	Caffeic Acid and Resveratrol	*Drosophila melanogaster*	MJD/SCA3	- improved survival and locomotor activity and decreased mutant ataxin-3 and ROS levels in tBH-treated SCA3 Drosophila [115]
	Aqueous root extract from swallowroot (*Decalepis Hamiltonii),* ellagic acid		PD	- significantly improved climbing ability and circadian rhythm of locomotor activity, reduced levels of ROS and LPO and enhanced catalase (CAT) and superoxide dismutase (SOD) activity [78]
	Peacocks tail (brown algae; *Padina pavonica*) and barbary fig (*Opuntia ficus-indica*)		AD	- improvement of the survival and mobility of AD models, *Padina pavonica* extracts improved a pan-neuronal expression of a double dose of Aβ42 (late-onset AD model), *Opuntia ficus-indica* showed positive effects in a model of early onset AD (flies expressing the Arctic Aβ42)
	Sinapic acid, Sodium sinapate	Mice	AD, dementia	- rescued neuronal cell death and attenuated the increase of iNOS expression, glial cell activations and nitrotyrosine expressions induced by Aβ1–42 protein, attenuated memory impairment as well as cerebral protective and cognition-improving effects [118,119]
	Grape seed polyphenol extract		AD	- attenuated the development of tau neuropathology in a TMHT mouse model of AD through mechanisms associated with attenuation of extracellular signal-receptor kinase 1/2 signaling in the brain and interference with the assembly of tau peptides into neurotoxic aggregates [120]
	Epigallocatechin Gallate (EGCG, polyphenol in green tea)		PD	- regulation of the iron-export protein ferroportin in substantia nigra by EGCG, reduction of oxidative stress and neurorescue effect against MPTP-induced functional and neurochemical deficits in mice [121]
	Korean black soybeans/ anthocyanins		AD	- regulation of the PI3K/Akt/GSK3 pathway, activation of the downstream endogenous anti-oxidant Nrf2 transcription factor and its target genes HO-1 and GCLM led to amyloid β oligomer (AβO)-induced elevation of ROS was reduced and neurodegeneration prevented [94]
	resveratrol		MJD/SCA3	- activation of the histone deacetylase enzyme SIRT1 pathway, showing improvement in motor behaviour when treating animals at a post symptomatic stage of disease development [97]
	Ferulic acid	Rats	PD	- rescued dopamine neurons in substantia nigra pars compacta area and nerve terminals in the striatum from the rotenone insult; restored antioxidant enzymes, prevented depletion of glutathione, and inhibited lipid peroxidation and attenuation of microglial and astrocytic activation [104]
	Sinapic acid		PD	- significantly improved turning behavior, prevented loss of dopaminergic neurons in substantia nigra pars compacta, lowered iron reactivity, and attenuated level of malondialdehyde and nitrite [106]

MPP^+^-1-methyl-4-phenylpyridinium; MTPT- 1-methyl-4-phenyl-1,2,3,6-tetrahydropyridine, prodrug of MPP^+^; 6-OHDA-6-hydroxydopamine.

## Abbreviations

6-OHDA	6-hydroxydopamin
AAPH	2,2′-Azobis(2-amidinopropane) dihydrochloride
ABTS	2,2′-azino-bis(3-ethylbenzothiazoline-6-sulphonic acid)
AChE	Acetylcholinesterase
AD	Alzheimer’s disease
ADAS-Cog	AD Assessment Scale cognitive subscale
ADL	activities of daily living
AEP	asparagine endopeptidase
ALS	Amyotrophic lateral sclerosis/MND motor neurone disease
APP	amyloid precursor protein
APP/PS1	AD mouse model
Aβ	amyloid beta
α-syn	alpha-synuclein
CAT	catalase
CCl_4_	carbon tetrachloride
CHP	cumene hydroperoxide
CRISP	clustered regularly interspaced short palindromic repeats
DPPH	2,2-diphenylpicrylhydrazyl
ERK	extracellular signal-regulated kinase
FDA	Food and Drug Administration
FTLD	frontotemporal lobar degeneration
GAE	gallic acid equivalence
GCLM	Glutamate-cysteine ligase regulatory subunit
GFP	green fluorescence protein
GSH	glutathione
HCH	hexachlorocyclohexane
HD	Huntington’s disease
HO-1	heme oxygenase-1
HPLC	high performance liquid chromatography
L-DOPA	Levodopa
LRRK2	leucine-rich repeat kinase-2
MAPK	mitogen-activated protein kinase
MDA	malondialdehyde
MJD	Machado-Joseph disease
SCA3	Spinocerebellar ataxia type 3
MPP^+^	1-methyl-4-phenylpyridinium
MS	mass spectrometry
NCCIH	National Centre for Complementary and Integrative Health
NGF	nerve growth factor
NMDA	N-Methyl-D-aspartate
NMR	nuclear magnetic resonance (spectroscopy)
Nrf2	Nuclear factor (erythroid-derived 2)-like 2
PD	Parkinsn’s disease
PI3K/Akt/GSK3	Phosphoinositide 3-kinase/RAC-alpha serine/threonine-protein kinase/Glycogen synthase kinase 3
PKC	protein kinase C
RNAi	RNA interference
RNS	reactive nitrogen species
ROS	reactive oxygen species
SCA	Spinocerebellar ataxia
SIRT1	sirtuin (silent mating type information regulation 2 homolog) 1 (*S. cerevisiae*)
SOD	superoxide dismutase
tBH	tert-butyl-hydroperoxide
UV	ultra violet
